# Effects of Sodium-Glucose Co-transporter 2 Inhibitors on Hemoglobin Levels: A Meta-analysis of Randomized Controlled Trials

**DOI:** 10.3389/fphar.2021.630820

**Published:** 2021-03-12

**Authors:** Wei Qu, Li Yao, Xiaodan Liu, Tianhua Xu, Binyao Tian

**Affiliations:** Department of Nephrology, the First Hospital of China Medical University, Shenyang, China

**Keywords:** meta-analysis, SGLT2 inhibitors, type 2 diabetes, chronic kidney disease, hemoglobin

## Abstract

**Background:** This study aimed to explore the effects of sodium-glucose co-transporter 2 (SGLT2) on hemoglobin levels in patients with type 2 diabetes mellitus (T2DM) and chronic kidney disease.

**Methods:** PubMed, EMBASE, the Cochrane Central Register of Controlled Trials, the China National Knowledge Infrastructure database, Wanfang Digital Periodicals Database (WFDP) and the Chinese Biological and Medical database (CBM) were searched for randomized trials of SGLT2 inhibitors in patients with T2DM and chronic kidney disease up to July 25, 2020. A total of four studies that included 19,259 patients were identified.

**Results:** Compared to control patients, SGLT2 inhibitors were shown to increase hemoglobin levels in patients with T2DM and chronic kidney disease (standard mean difference = 0.70, 95% CI, 0.59–0.82, *p* < 0.0001).

**Conclusion:** SGLT2 inhibitors may bring additional benefits to patients with T2DM and chronic kidney disease.

## Introduction

In the past ten years, the incidence of type 2 diabetes mellitus (T2DM) has been increasing (International Diabetes Federation, 2017) which indicates a massive increase in end-stage renal disease on a global scale. One of the most common complications of chronic kidney disease is renal anemia (Sugahara et al., 2017). The presence of anemia significantly increases the risk of micro- and macrovascular complications. Patients who are not properly treated have significantly reduced quality of life and a poor prognosis (Sugahara et al., 2017).

Sodium-glucose co-transporter 2 (SGLT2) inhibitors are a newly approved class of oral hypoglycemic agents that increase the excretion of glucose in the urine by inhibiting the reabsorption of urine glucose in the proximal tubules of the kidney, thereby reducing blood glucose levels, weight and blood pressure (Polidori et al., 2014; Inagaki et al., 2015). In addition, SGLT2 inhibitors also have a protective effect on the kidney (Xu et al., 2017) as evidence has shown that after treatment with SGLT2, hemoglobin levels are increased (Maruyama et al., 2019).

In this study, we aimed to ascertain the effects of SGLT2 inhibitors on the hemoglobin levels in patients with T2DM and chronic kidney disease.

## Methods

### Data Sources and Search Strategies

The PubMed, EMBASE, the Cochrane Central Register of Controlled Trials, the China National Knowledge Infrastructure (CNKI) database, Wanfang Digital Periodicals database (WFDP), the Chinese Biological and Medical database (CBM) were searched. The following Medical Subject Headings (MeSH) terms and free-text terms were applied: “Sodium-Glucose Transporter 2 Inhibitors”, “Sodium Glucose Transporter 2 Inhibitors”, “SGLT2 Inhibitors”, “SGLT-2 Inhibitors”, “SGLT 2 Inhibitors”, “Gliflozins”, “Renal Insufficiency, Chronic”, “Chronic Renal Insufficiencies”, “Renal Insufficiencies, Chronic”, “Chronic Renal Insufficiency”, “Kidney Insufficiency, Chronic”, “Chronic Kidney Insufficiency”, “Chronic Kidney Insufficiencies”, “Kidney Insufficiencies, Chronic”, “Chronic Kidney Diseases”, “Chronic Kidney Disease”, “Disease, Chronic Kidney”, “Diseases, Chronic Kidney”, “Kidney Disease, Chronic”, “Kidney Diseases, Chronic”, “Chronic Renal Diseases”, “Chronic Renal Disease”, “Disease, Chronic Renal”, “Diseases, Chronic Renal”, “Renal Disease, Chronic”, “Renal Diseases, Chronic”. All publications up to July 25, 2020 were selected without the restriction of origins, countries, languages or article types.

### Selection Standards

Published articles that were included in the analysis were required to meet the following criteria: 1) the eligible subjects were men and women with T2DM and chronic kidney disease; 2) interventions involved treatment with SGLT2 inhibitors alone or with other hypoglycemic agents; 3) studies compared placebo control or standard of care; 4) outcomes reported changes in hemoglobin levels from baseline; 5) studies that were randomized controlled trials (RCTs); 6) studies with follow-up times of 12 weeks or longer. Observational studies, non-randomized trials and uncontrolled trials were excluded from the analysis.

### Data Extraction and Quality Assessment

Two investigators extracted the following data independently from eligible publications: first author, publication year, study design, inclusion criteria, sample size, patient characteristics, interventions (types and doses of SGLT2 inhibitors), comparison (placebo control or standard care), follow-up duration and outcomes (changes in hemoglobin levels from baseline). The unit of hemoglobin levels was uniformly converted into g/l. Discrepancies were resolved by the discussion between two investigators. The Cochrane risk-of-bias tool was adopted to assess randomization, masking of treatment allocation, blinding, adherence and withdrawals for each of the RCTs (Higgins et al., 2011).

### Statistical Analysis

Data analysis was performed using Stata version 12.0 software. The effect sizes on scores were presented as the standard mean difference (SMD) and 95% confidence intervals (CIs). The Chi-squared test based on Q-statistic and I2 statistics was used to estimate the heterogeneity (I2 ≤ 25%, low heterogeneity; 25% < I2 < 50%, moderate heterogeneity; I2 ≥ 50%, high heterogeneity) (Higgins et al., 2003). A fixed-effects model was used to pool the results when heterogeneity was ≤50%, while a random-effects model was used when heterogeneity was >50% (Mantel and Haenszel, 1959; DerSimonian and Laird, 1986). A sensitivity analysis was performed to reveal the influence of a single study on the overall pooled estimates by deleting one study in each turn. Publication bias was evaluated using the Begg’s and Egger’s tests (Begg and Mazumdar, 1994; Egger et al., 1997). *p* values (<0.05) were considered to represent statistically significant publication bias.

## Results

### Description of the Studies

**FIGURE 1:**
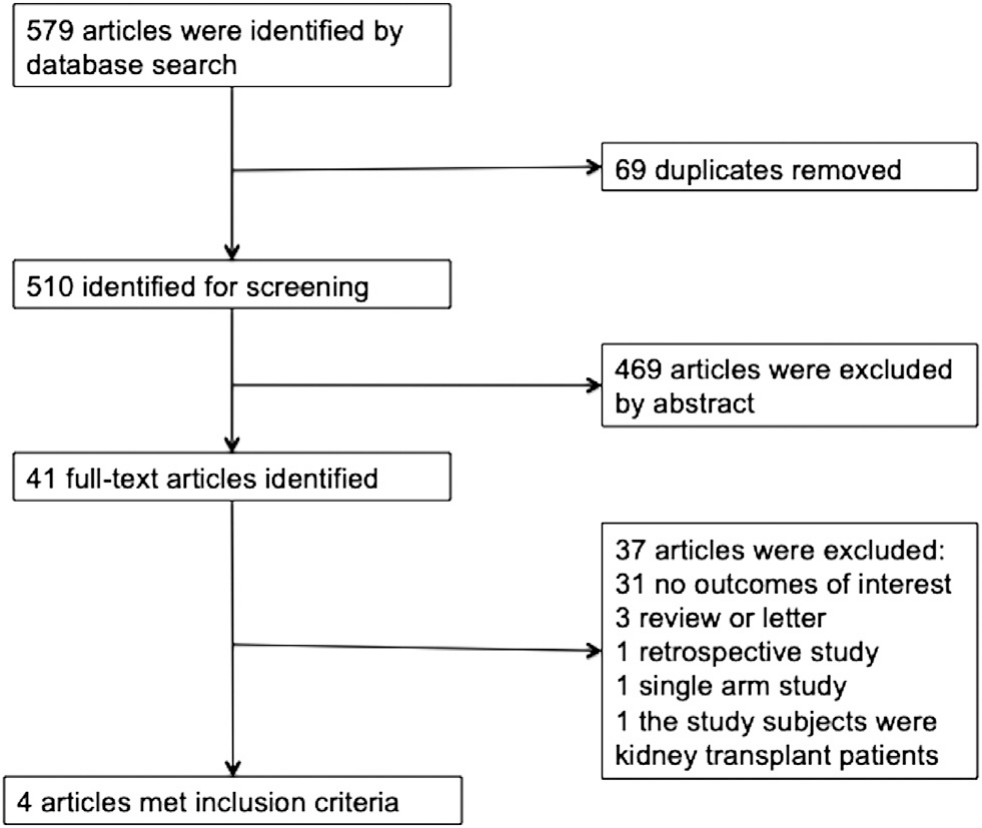
Eligibility of the studies for inclusion in the meta-analysis.

**FIGURE 2:**
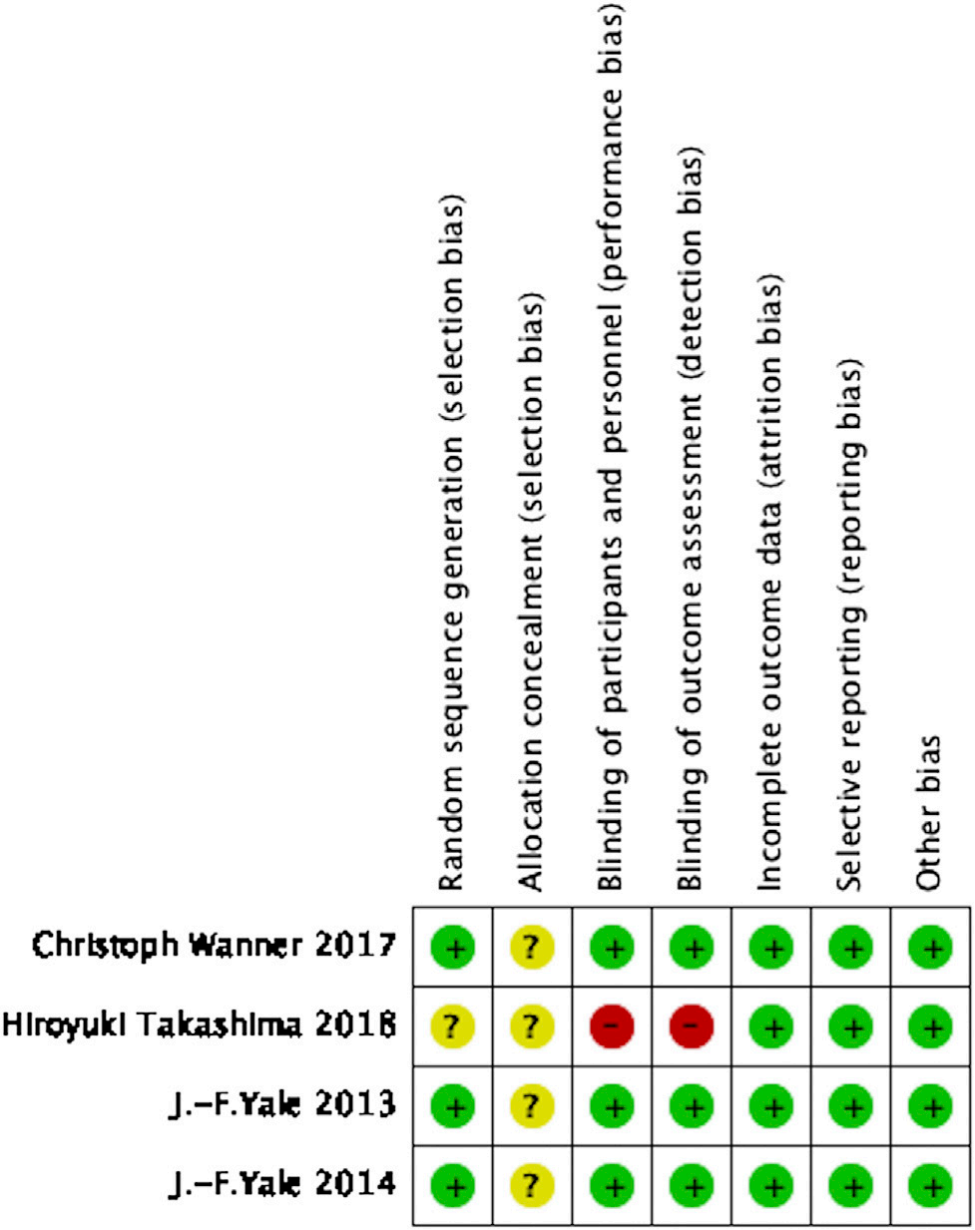
Risk of bias in the included studies.

A total of 579 references were retrieved and finally, four studies (Yale et al., 2013; Yale et al., 2014; Wanner et al., 2018; Takashima et al., 2018) met the inclusion criteria for the meta-analysis (Figure 1). The sample size included 19,259 patients with T2DM and chronic kidney disease in this meta-analysis (Table 1 Characteristics of the included studies). Three of the studies were multi-center, double-blind, placebo-controlled trials (Yale et al., 2013; Yale et al., 2014; Wanner et al., 2018) in which white people took up the majority of patients and one was a single-center, open-label, parallel-group trial conducted in Japan (Takashima et al., 2018). The types of SGLT2 inhibitors included canagliflozin (100 mg/300 mg) and enpagliflozin (10 mg/25 mg). Follow-up duration ranged from 12 to 164 weeks. All included studies were evaluated in terms of the risk of bias using the Cochrane risk of bias tool and the details are illustrated in Figure 2 (Risk of bias in the included studies).

**TABLE 1:** Summary of the characteristics of the included studies.

First author, Year	RCT or not	Inclusion criteria	Age, Years (T/C)	SGLT2i dosing	Comparison	Period of treatment	Treatment group			Control group		
							Sample size (n)	Hemoglobin outcome (M ± SD)	Hematocrit outcome (M±SD)	Sample size (n)	Hemoglobin outcome (M ± SD)	Hematocrit Outcome (M±SD)
Yale et al. (2013)	Y	T2DM; HbA1c ≥ 7.0, while ≤10.5%; eGFR ≥30, while <50 ml/min/1.73 m^2^	69.5/68.2	Canagliflozin 100 mg QD	Placebo control	26-weeks	69	5.3 ± 7.4	6 ± 7.6	62	−0.5 ± 8.1	−0.1 ± 9.1
		T2DM; HbA1c ≥ 7.0, while ≤10.5%; eGFR ≥30, while <50 ml/min/1.73 m^2^	67.9/68.2	Canagliflozin 300 mg QD	Placebo control	26-weeks	76	3.1 ± 5.9	4.8 ± 6.9	62	−0.5 ± 8.1	−0.1 ± 9.1
Yale et al. (2014)	Y	T2DM; HbA1c ≥ 7.0, while ≤10.5%; eGFR ≥30, while <50 ml/min/1.73 m^2^	69.5/68.2	Canagliflozin 100 mg QD	Placebo control	52-weeks	62	6.5 ± 7.9	6.6 ± 8.5	57	−1.4 ± 7.8	−0.9 ± 8.4
		T2DM; HbA1c ≥ 7.0, while ≤10.5%; eGFR ≥30, while <50 ml/min/1.73 m^2^	67.9/68.2	Canagliflozin 300 mg QD	Placebo control	52-weeks	70	4.2 ± 9.6	5.9 ± 10.9	57	−1.4 ± 7.8	−0.9 ± 8.4
Wanner et al. (2017)	Y	T2DM; HbA1c ≥ 7.0, while ≤10%; eGFR ≥30, while <60 ml/min/1.73 m^2^	66.2/66.0	Empagliflozin 10 mg QD	Placebo control	12-weeks	605	5.7 ± 7.4	——	607	−0.5 ± 7.4	——
		T2DM; HbA1c ≥ 7.0, while ≤10%; eGFR ≥30, while <60 ml/min/1.73 m^2^		Empagliflozin 25 mg QD	Placebo control	12-weeks	607	6.3 ± 7.4	——	607	−0.5 ± 7.4	——
		T2DM; HbA1c ≥ 7.0, while ≤10%; eGFR ≥30, while <60 ml/min/1.73 m^2^	66.2/66.0	Empagliflozin 10 mg QD	Placebo control	164- week	605	6.2 ± 14.8	——	607	−2 ± 14.8	——
		T2DM; HbA1c ≥ 7.0, while ≤10%; eGFR ≥30, while <60 ml/min/1.73 m^2^		Empagliflozin 25 mg QD	Placebo control	164- week	607	5.4 ± 14.8	——	607	−2 ± 14.8	——
		T2DM; HbA1c ≥ 7.0, while ≤10%; eGFR ≥60 ml/min/1.73 m^2^	61.6/61.9	Empagliflozin 10 mg QD	Placebo control	12- week	1740	6 ± 8.3	——	1726	0 ± 8.3	——
		T2DM; HbA1c ≥ 7.0, while ≤10%; eGFR ≥60 ml/min/1.73 m^2^		Empagliflozin 25 mg QD	Placebo control	12- week	1733	7 ± 8.3	——	1726	0 ± 8.3	——
		T2DM; HbA1c ≥ 7.0, while ≤10%; eGFR ≥60 ml/min/1.73 m^2^	61.6/61.9	Empagliflozin 10 mg QD	Placebo control	164- week	1740	5.3 ± 16.7	——	1726	−2.3 ± 16.6	——
		T2DM; HbA1c ≥ 7.0, while ≤10%; eGFR ≥60 ml/min/1.73 m^2^		Empagliflozin 25 mg QD	Placebo control	164- week	1733	5.9 ± 16.7	——	1726	−2.3 ± 16.6	——
Takashima et al. (2018)	Y	T2DM; HbA1c <10.0%; eGFR ≥45, while <90 ml/min/1.73 m^2^	64.7/65.4	Canagliflozin 100 mg QD	Usual care	52-weeks	21	8 ± 6	——	21	−2 ± 3	——

T2DM, type 2 diabetes mellitus; eGFR, estimated glomerular filtration rate; RCT, randomized controlled trial; C, control group; T, treatment group; QD, once a day; SGLT2i, sodium glucose co-transporter 2 inhibitors; HbA1c, hemoglobin A1c; M ± SD, mean ± standard deviation.

### Risk of Bias

With the exception of one open-label study, the other three studies on random sequence generation were fully considered. The studies were all double-blind trials but there was no further explanation on the details of allocation concealment. There were no incomplete outcomes and selective reporting in the four studies. Based on the characteristics, we believe that the included studies had a low risk of bias.

### Effects of Interventions on Hemoglobin Levels

**FIGURE 3:**
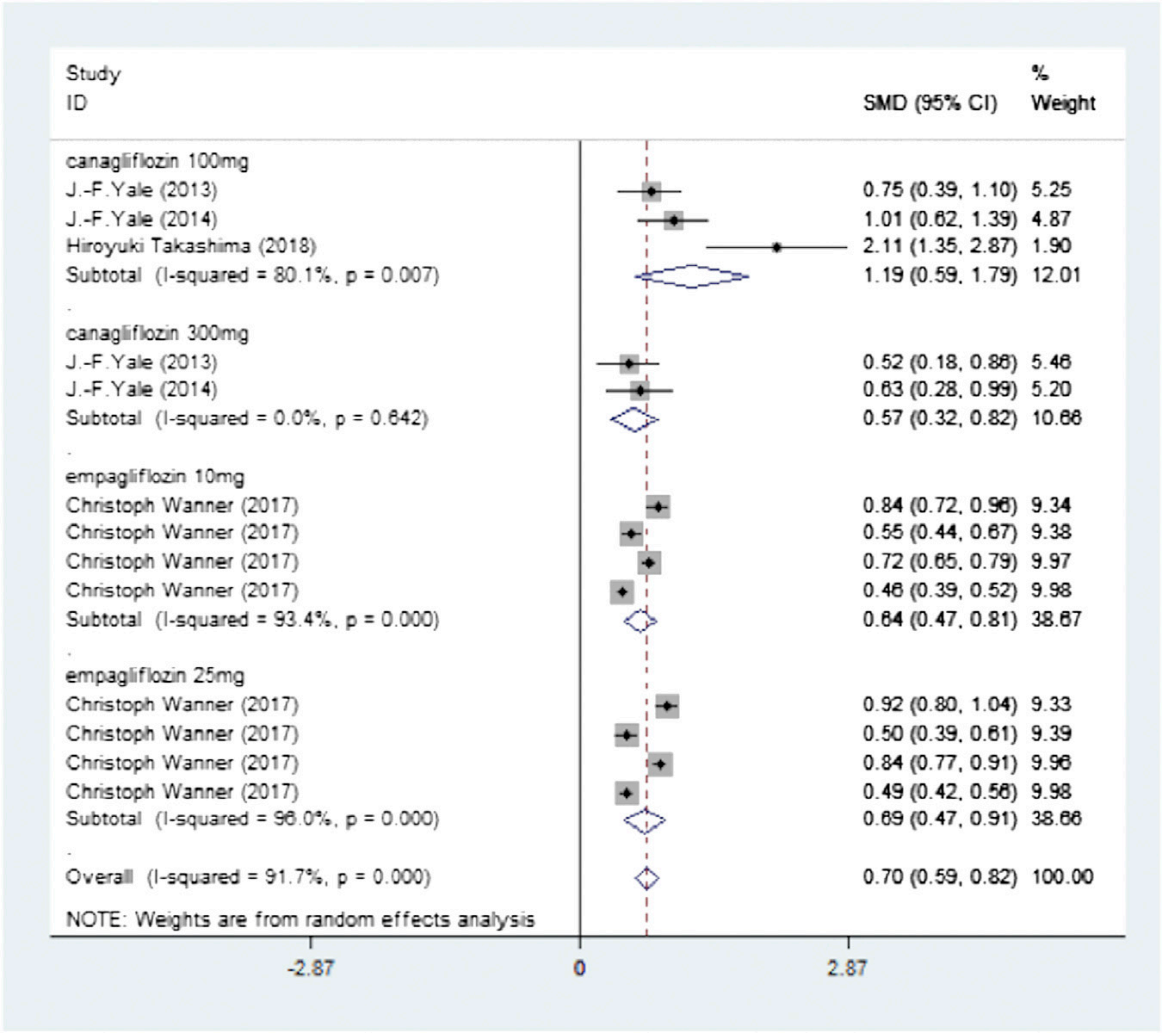
Meta-analysis and forest plot of hemoglobin levels in the experimental and the control groups.

Four studies (Four publications) investigated a total of 19,259 participants (experimental group: 9,668, control group: 9,591) and reported hemoglobin levels. There was high heterogeneity (I2 = 91.7%, *p* < 0.0001) and so the random-effects model was used. The pooled effect size showed significant differences in hemoglobin levels (SMD = 0.70, 95% CI, 0.59–0.82, *p* < 0.0001) in favor of the experimental groups compared to the control groups (Figure 3 Meta-analysis and forest plot of hemoglobin levels for experimental group compared with the control group).

### Effects of Interventions on Hematocrit

**FIGURE 4:**
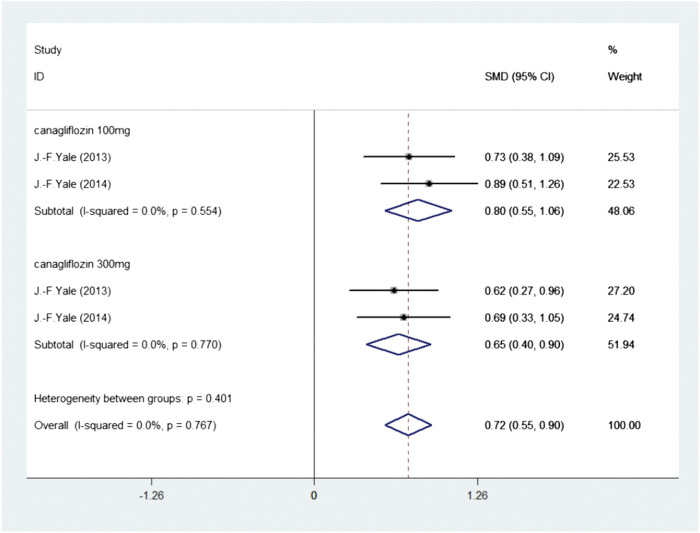
Meta-analysis and forest plot of Hematocrit for experimental group compared with control group.

We further analyzed the effect of SGLT2 inhibitors on hematocrit. Two publications investigated 515 participants (experimental group: 277, control group: 238) reported Hematocrit. There was no heterogeneity (I2 = 0, *p* = 0.767); thus, the fixed-effects model was used. The pooled effect size showed a significant difference in Hematocrit (SMD = 0.72, 95%CI 0.55–0.90, *p* < 0.05) in favor of experimental group, compared with the control group (Figure 4 Meta-analysis and forest plot of Hematocrit for experimental group compared with control group).

### Subgroup Analysis

**FIGURE 5:**
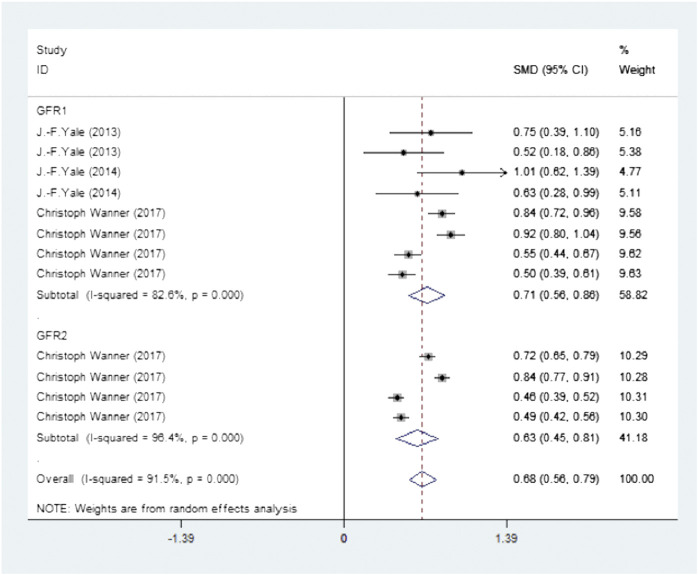
Meta-analysis and the forest plot data of hemoglobin levels in the subgroup analysis.

To explore the sources of heterogeneity, we conducted a subgroup analysis of the type and dosage of SGLT2 inhibitors and eGFR. According to the type and dosage of the drug, treatments were divided into four subgroups (canagliflozin 100 mg, canagliflozin 300 mg, empagliflozin 10 mg and empagliflozin 25 mg). Subgroups were divided as to eGFR based on 30 ≤ eGFR <60 ml/min/1.73 m^2^ and eGFR ≥60 ml/min/1.73 m^2^. The results are summarized in Figure 5. All of the results in the subgroups were statistically significant compared to those in the control group, however, heterogeneity was not significantly reduced following subgroup analysis.

### Sensitivity Analysis

A sensitivity analysis was conducted by sequentially removing one study to observe the influence of each of the included studies on the overall pooled SMD. No single study was found to significantly influence the overall pooled SMD indicating that the results were stable.

### Publication Bias

In assessing publication bias, a funnel plot for the four studies analyzed was constructed. The shape of the funnel plot was symmetrical indicating the absence of publication bias. No significant bias was observed using the Begg’s rank correlation test (Z = 1.4, *p* = 0.161 (>0.05)) and Egger’s linear regression test (t = 0.78, *p* = 0.451 (>0.05)).

## Discussion

This article conducted a meta-analysis of four randomized controlled studies to explore the effects of SGLT2 inhibitors on hemoglobin levels in diabetic patients with chronic kidney disease. The results showed that the hemoglobin levels of patients after treatment with SGLT2 inhibitors increased from baseline and the differences were statistically significant. The hematocrit levels of patients after treatment with SGLT2 inhibitors increased from baseline and the differences were statistically significant. Whether it was different type and dosage of the drug (canagliflozin 100 mg, canagliflozin 300 mg, empagliflozin 10 mg and empagliflozin 25 mg), or different eGFR (30 ≤ eGFR <60 ml/min/1.73 m^2^ and eGFR ≥60 ml/min/1.73 m^2^), the differences were statistically significant.

### Comparison With Other Published Studies

Most of the observations from previous reports in the literature and meta-analysis demonstrate the effects of SGLT2 on blood sugar levels, cardiovascular events and renal outcomes. There are very few studies that have analyzed the effects of SGLT2 inhibitors on hemoglobin levels or have performed meta-analyses of these effects across multiple RCTs.

### Mechanisms

SGLT2 inhibitors protect patients with T2DM and chronic kidney disease through several different mechanisms. First, in diabetic patients, upregulation of SGLT2 increases the reabsorption of sodium and glucose by the proximal tubules, SGLT2 inhibitors lower blood sugar by blocking the glucose reabsorption of SGLT2 in the proximal renal tubules. Second, SGLT2 inhibitors also have a certain effect on renal hemodynamics. SGLT2 inhibitors block the reabsorption of glucose and sodium in the proximal tubules and increase the transport of sodium to the macula densa, thereby restoring impaired tubuloglomerular feedback. Thus, SGLT2 inhibitors can alleviate glomerular filtration in the early stage of diabetic nephropathy, reduce albuminuria, and delay the decline of renal function for a long time (Cherney et al., 2014; Škrtić and Cherney, 2015). Besides, the protective effects of SGLT2 inhibitors are also manifested in the reduction of blood pressure, weight loss, osmotic diuresis, reduction of inflammation, fibrosis, and proliferation of proximal renal tubular cells (Panchapakesan et al., 2013).

The mechanisms by which SGLT2 inhibitors improve hemoglobin levels in patients with diabetes and chronic kidney disease are not fully understood. It has been reported that SGLT2 inhibitors have diuretic-like effects and reduce plasma volume (Lambers Heerspink et al., 2013). It has also been reported that in diabetic patients with normal renal function, SGLT2 inhibitors can reduce the load caused by excessive glucose reabsorption in the proximal tubules, and can improve renal tubular interstitial hypoxia and restore fibroblasts to produce erythropoietin (EPO) causing hemoglobin levels to increase (Lambers Heerspink et al., 2013). In diabetic patients with chronic kidney disease, SGLT2 inhibitors can also increase hemoglobin levels by promoting the production of EPO. Studies have also shown that SGLT2 inhibitors can upregulate AMPK and SIRT1 (Swe et al., 2019; Packer 2020), thereby inhibiting HIF-1α and activating HIF-2α (Treins et al., 2006; Dioum et al., 2009; Lim et al., 2010). HIF-2α is the isoform responsible for the synthesis of EPO (Eckardt and Kurtz, 2005). The increase in hematocrit may be due to the decrease in plasma volume caused by SGLT2 inhibitor-related diuresis and natriuresis, or it may be due to increased erythropoiesis after the treatment of SGLT2 inhibitor. The increase in hematocrit during treatment with SGLT2 inhibitors may indicate the improvement of hypoxia and oxidative stress in the tubular interstitial area of the renal cortex, as well as the recovery of EPO production by interstitial fibroblast-like cells. SGLT2 inhibitors also inhibit hepcidin, which may lead to increased iron bioavailability and utilization and increased red blood cell production (Ghanim et al., 2020). These effects on erythropoiesis suggest that SGLT2 inhibitors may reduce the incidence of anemia. The post-hoc analysis of the CREDENCE trial by Megumi Oshima et al. found that the risk of anemia or the risk of starting anemia treatment in the anemia group of patients with type 2 diabetes and chronic kidney disease was significantly lower than that of the placebo group (Oshima et al., 2020). In the exploratory analysis of EMPA-REG test data, the increase in hematocrit during empagliflozin treatment was closely related to beneficial cardiovascular outcomes (Inzucchi et al., 2018). Studies have shown that increased expression of HIF-2α in cardiomyocytes can protect mitochondrial integrity and prevent experimental ischemic damage (Bautista et al., 2009; Mastrocola et al., 2016). For the same blood flow, a higher hematocrit is expected to deliver more oxygen to the tissue (Testani et al., 2010). It has been suggested that the increase in hematocrit may contribute to the cardioprotective effect of these drugs by increasing the oxygen-carrying capacity (Ferrannini et al., 2016; Lytvyn et al., 2017).

### Limitations

Several limitations of this study should be noted. Firstly, a total of four articles were included in the analysis which is a small sample size, however, the total number of patients included was nor large. Secondly, the majority of the subjects were Caucasian and so the applicability of the data to other races including Asians requires further investigation. Thirdly, although we concluded that hemoglobin levels increased after treatment with SGLT2 inhibitors, we did not observe differences in the effects of different types of SGLT2 inhibitors on hemoglobin levels, the relationship between the increase in hemoglobin level and the duration of medication. Fourthly, among the populations included in the study, some had eGFR ≥ 60 ml/min/1.73 m^2^. This meant that a small number of patients with normal renal function may have been included. In addition, the population included in the study did not have obvious renal anemia before SGLT2 inhibitors treatment. Therefore, for patients with significant renal anemia, the benefits of SGLT2 inhibitors need to be further investigated.

In summary, patients with T2DM and chronic kidney disease have increased hemoglobin and hematocrit levels after treatment with SGLT2 inhibitors. SGLT2 inhibitors may bring additional benefits to patients with T2DM and chronic kidney disease.

## Data Availability

The original contributions presented in the study are included in the article/Supplementary Material, further inquiries can be directed to the corresponding author.
